# RUNX1/NPM1/H3K4me3 complex contributes to extracellular matrix remodeling via enhancing FOSL2 transcriptional activation in glioblastoma

**DOI:** 10.1038/s41419-024-06481-4

**Published:** 2024-01-29

**Authors:** Xiaoteng Cui, Dawei Huo, Qixue Wang, Yunfei Wang, Xiaomin Liu, Kai Zhao, Yongping You, Junxia Zhang, Chunsheng Kang

**Affiliations:** 1 Lab of Neuro-oncology, Tianjin Neurological Institute, Tianjin Medical University General Hospital, Key Laboratory of Post-Neuro Injury Neuro-repair and Regeneration in Central Nervous System, Ministry of Education and Tianjin City, Tianjin, 300052 China; 2 Bone Marrow Transplantation Center, the First Affiliated Hospital, Zhejiang University School of Medicine, Liangzhu Laboratory, Institute of Hematology, Zhejiang University, Zhejiang Province Engineering Laboratory for Stem Cell and Immunity Therapy, Hangzhou, 310003 China; 3 Neuro-Oncology Center, Tianjin Huanhu Hospital, Nankai University, Tianjin, 300350 China; 4 Department of Neurosurgery, the Affiliated Hospital of Qingdao University, Qingdao, 266000 China; 5 Department of Neurosurgery, The First Affiliated Hospital of Nanjing Medical University, Institute for Brain Tumors, Jiangsu Collaborative Innovation Center for Cancer Personalized Medicine, Nanjing Medical University, Nanjing, 210029 China

**Keywords:** CNS cancer, Cancer microenvironment

## Abstract

Extracellular matrix (ECM) remodeling has been implicated in the tumor malignant progression and immune escape in glioblastoma (GBM). Runt-related transcription factor 1 (RUNX1) is a vital transcriptional factor for promoting tumorigenesis and invasion in mesenchymal subtype of GBM. But the correlation between RUNX1 and ECM genes expression and regulatory mechanism of RUNX1 on ECM genes expression remain poorly understood to date. In this study, by using integral analysis of chromatin immunoprecipitation-sequencing and RNA sequencing, we reported that RUNX1 positively regulated the expression of various ECM-related genes, including Fibronectin 1 (FN1), Collagen type IV alpha 1 chain (COL4A1), and Lumican (LUM), in GBM. Mechanistically, we demonstrated that RUNX1 interacted with Nucleophosmin 1 (NPM1) to maintain the chromatin accessibility and facilitate FOS Like 2, AP-1 Transcription Factor Subunit (FOSL2)-mediated transcriptional activation of ECM-related genes, which was independent of RUNX1’s transcriptional function. ECM remodeling driven by RUNX1 promoted immunosuppressive microenvironment in GBM. In conclusion, this study provides a novel mechanism of RUNX1 binding to NPM1 in driving the ECM remodeling and GBM progression.

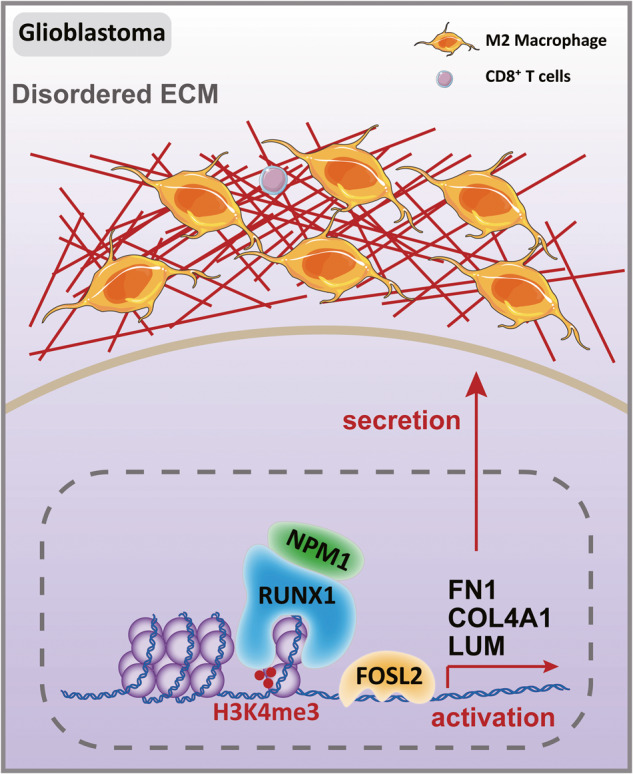

## Introduction

Glioblastoma (GBM) is the most common and lethal primary malignancy in the central nervous system (CNS). The intrinsic resistance to the radio and chemotherapy, as well as frequent incidences of post-surgical recurrence, are the major characteristics of GBMs [1, 2]. Therefore, outcomes of conventional treatment options, including surgical resection combined with radiotherapy and/or chemotherapy remain unsatisfactory [3]. Due to its primary location in the immune-privileged brain region and the existence of the blood-brain barrier (BBB), GBM possesses a unique tumor immune microenvironment (TIME) involving a large number of infiltrating macrophages/microglia and fewer antitumor T-cells, which renders the GBM unresponsive to immune-checkpoint therapeutics [4–6]. Therefore, unveiling the mechanism of the constitution of TIME in GBM patients could be highly important for developing GBM-targeted therapies.

An extracellular matrix (ECM) is a non-cellular connective tissue that exists in the interstitial space of cells and tissues in highly complex and ordered manners. ECM is mainly composed of structural glycoproteins (collagens, fibronectin, etc.), leucine-rich repeat proteoglycans [such as Lumican (LUM)], and glycosaminoglycans (such as hyaluronic acid) [7]. Physiological interactions among these ECM components themselves and with cells around are crucial for supporting the tissue structure, morphology, and function [8]. The brain ECM contains a small amount of fibrin but a large number of proteoglycan molecules wrapping around neurons and glial cells. GBM pathology can alter or remodel the ECM composition, resulting in significantly increasing relative volume fractions, which in turn facilitates the compactness of TIME [9]. Increased LUM secretion is important for the regulation and correction of collagen bundle formation. On the other hand, ECM remodeling plays a vital role in the TIME by activating the integrin signaling [10]. However, the underlying regulatory mechanism of ECM remodeling in GBM pathogenesis remains unclear.

Runt-related transcription factor 1 (RUNX1), also known as acute myeloid leukemia 1 (AML1), is a key modulator of developmental hematopoiesis, as well as a critical transcription factor (TF) for various hematopoietic genes coding for granulocyte-macrophage colony-stimulating factor (GM-CSF) and myeloperoxidase (MPO) [11]. RUNX1 heterodimerizes with core-binding factor subunit beta (CBFβ) to bind the consensus TGTGGT DNA motif via its Runt domain and regulates the gene expression of downstream factors [12]. Interestingly, chromosomal translocations of RUNX1 and/or CBFβ are frequently detected in most hematological tumors [13]. Ro5-3335, a small-molecule inhibitor (SMI) developed to target the RUNX1-CBFβ interaction, can treat leukemia by blocking the RUNX1/CBFβ transactivation [14]. In GBM, aberrant RUNX1 expression and activity are vital for the progression of malignancy [15, 16]. We have shown that RUNX1 can be a potent biomarker for mesenchymal GBM [17]. However, the molecular mechanism of RUNX1 in facilitating the malignant progression of GBM remains mysterious.

In this study, we reported a significantly positive correlation between RUNX1 and the expression of important ECM genes, including *fibronectin 1* (*FN1*), *collagen type IV alpha 1 chain* (*COL4A1*), and *LUM* in the pathogenesis of GBM. RUNX1 was found to promote the expression of ECM genes and the extracellular secretion of their coding proteins in a TF-independent manner. Mechanistically, RUNX1 interacts with nucleophosmin (NPM1) to maintain chromatin accessibility and histone H3K4me3 (tri-methylation at K4) modification status in the promoter regions of ECM genes for their transcriptional activation through FOS-Like 2, AP-1 Transcription Factor Subunit (FOSL2). In vivo experiments further confirmed RUNX1’s function on promoting the TIME formation through ECM remodeling in GBM. Therefore, this study provides evidence for a novel mechanism of RUNX1 in the context of GBM pathogenesis through ECM remodeling.

## Results

### RUNX1 positively regulates the expression of ECM-related genes in GBM

**Fig. 1:**
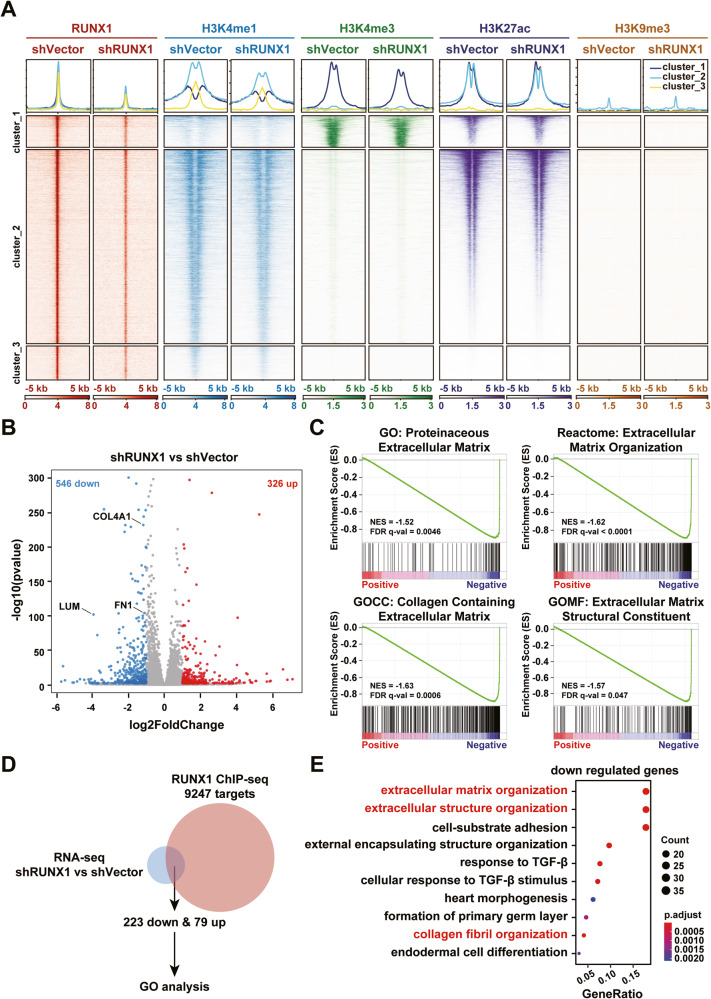
RUNX1 regulates the expression of ECM-related genes in GBM. **A** Fragmented chromatin fractions were immunoprecipitated with anti-RUNX1, anti-H3K4me, anti-H3K4me3, anti-H3K27ac, and anti-H3K9me3 antibodies and sequenced in shVector or shRUNX1-treated N9 cells. **B** Differentially expressed genes (DEGs) after RUNX1 knockdown (KD) in N9 cells were identified by RNA-sequencing and visualized as a volcano map. **C** Gene Set Enrichment Analysis (GSEA) analysis of downregulated genes in N9 shRUNX1 cells and compared with shVector cells. **D** Venn diagram of DEGs by RNA-sequencing and RUNX1-targeted genes by ChIP-sequencing showed that 302 genes coexisted in both the groups. **E** Gene ontology (GO) analysis was performed by using 223 downregulated genes.

Our previous study has revealed that RUNX1 expression is vital for GBM tumorigenesis. However, the actual underlying mechanism illustrating RUNX1-mediated gene expression regulation in GBM remains enigmatic. To identify the chromatin occupancy of RUNX1 in GBM tumor cells, we performed chromatin immunoprecipitation (ChIP) followed by sequencing (ChIP-seq) analysis in N9 cells stably infected with lentivirus carrying shRNA targeting RUNX1 or shVector. Compared to the shVector group, RUNX1-enriched peaks were significantly decreased in the shRUNX1 group, indicating the specificity of RUNX1’s chromatin enrichment (Fig. 1A). Peak annotation results showed that among the RUNX1-immunoprecipitated peaks, nearly 25% of peaks were correlated with the promoter regions, 28% were from distal intergenic regions, and the rest were distributed across intragenic regions (Supplementary Fig. 1A), suggesting that transcriptional activation is not the only way of RUNX1 to regulate downstream genes. At the same time, we interrogated the effect of RUNX1 knockdown (KD) on the histone modification using ChIP-seq analysis. As shown in Fig. 1A, levels of monomethylated and trimethylated forms of histone H3 at K4 (H3K4me1 and H3K4me3) were specifically reduced in RUNX1 KD cells, while levels of the acetylation of histone H3 at K27 (H3K27ac) and trimethylation at K9 (H3K9me3) were non-significantly affected. These results demonstrated that RUNX1 can regulate the level of gene expression activating histone modifications, especially H3K4me1 and H3K4me3.

Next, we employed an RNA sequencing (RNA-seq) approach to identify RUNX1-regulated genes in GBM. Through a series of stringent selection criteria, 326 upregulated and 546 downregulated genes were detected in shRUNX1 cells, compared to shVector cells (Fig. 1B). Gene-set enrichment analysis (GSEA) exhibited that RUNX1 KD was negatively correlated with ECM formation and organization (Fig. 1C). We then integrated the ChIP-seq and RNA-seq data to identify the downstream target genes in the RUNX1 regulatory pathway in GBM and detected 223 down- and 79 upregulated genes (Fig. 1D). Gene ontology (GO) analysis revealed that downregulated genes were significantly enriched in the ECM-associated cellular processes, while upregulated genes were mostly enriched in the MAPK pathway (Fig. 1E, Supplementary Fig. 1B).

Taken together, these results suggest that RUNX1 can promote the levels of H3K4me1 and H3K4me3, resulting in elevated expression of ECM-related genes in GBM.

### RUNX1 promotes the expression of ECM-related genes in a TF-independent manner

**Fig. 2:**
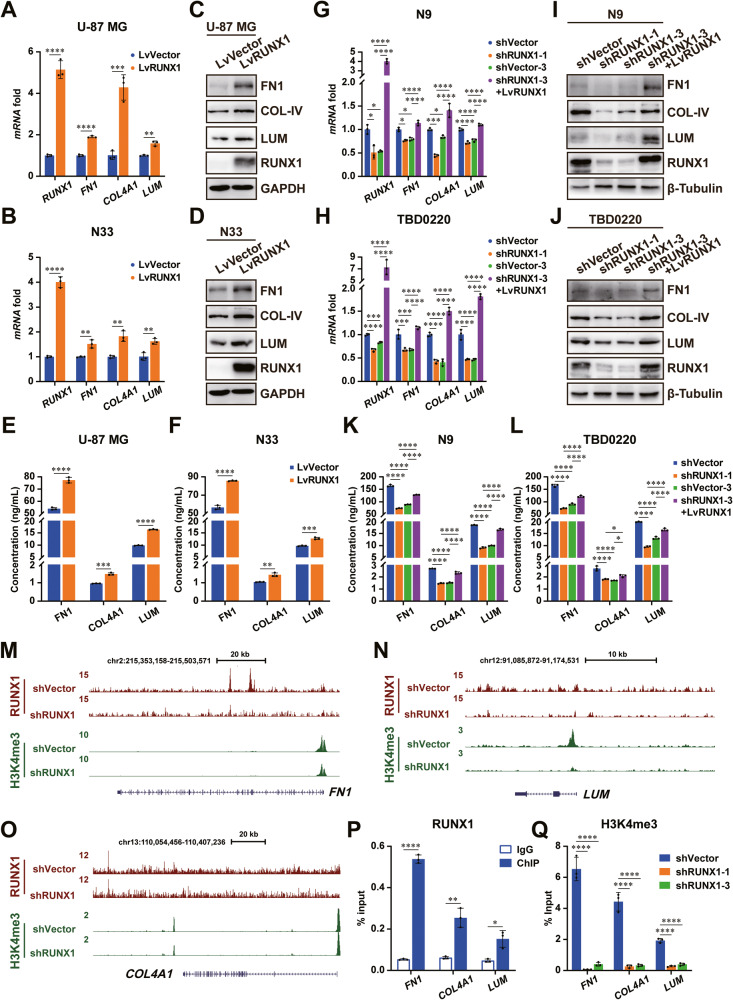
RUNX1 induces the expression of FN1, COL4A1, and LUM in GBM. **A**, **B** The mRNA levels of *RUNX1*, *FN1*, *COL4A1*, and *LUM* in U-87 MG and N33 cells with RUNX1 OE were respectively detected by qRT-PCR analyses. *GAPDH* was used as the internal control. **C**, **D** The protein levels of RUNX1, FN1, COL4A1, and LUM in U-87 MG and N33 cells with RUNX1 OE were respectively detected by WB analyses. GAPDH was used as the internal control. **E**, **F** The levels of FN1, COL4A1, and LUM in the supernatants of U-87 MG and N33 cells with RUNX1 OE were detected by ELISA assays. **G**, **H** The mRNA levels of *RUNX1*, *FN1*, *COL4A1*, and *LUM* in N9 and TBD0220 cells with RUNX1 KD and restored expression were respectively detected by qRT-PCR analyses. *GAPDH* was used as the internal control. **I, J** The protein levels of RUNX1, FN1, COL4A1, and LUM in N9 and TBD0220 cells with RUNX1 KD and restored expression were respectively detected by WB analyses. β-Tubulin was used as the internal control. **K**, **L** The levels of FN1, COL4A1, and LUM in the supernatants of N9 and TBD0220 cells with RUNX1 KD and restored expression were detected by ELISA assays. **M**–**O** Genomic snapshots of ChIP-sequencing analysis for RUNX1 and H3K4me3 in N9 cells transfected with shVector (upper track) or shRUNX1 (lower track). **P** ChIP analysis of the binding of RUNX1 to the promoter region of *FN1*, *COL4A1*, and *LUM* genes in N9 cells. **Q** H3K4me3 modifications in the promoter regions of *FN1*, *COL4A1*, and *LUM* genes were detected by ChIP-qRT-PCR method in RUNX1 KD N9 cells. Student’s t-test was performed for analyzing between two groups; one-way ANOVA was conducted for comparations of multiple groups. *P < 0.05, **P < 0.01, ***P < 0.001, ****P < 0.0001.

To investigate the mechanistic role of RUNX1 in regulating ECM-related genes expression in GBM, we overexpressed the expression of RUNX1 in U-87 MG and N33 cells via lentivirus containing recombinant RUNX1. Quantitative real-time polymerase chain reaction (qRT-PCR) analysis revealed that the expression of *FN1*, *COL4A1*, and *LUM* were significantly increased in LvRUNX1 groups, compared with those in LvVector groups (Fig. 2A, B). The protein levels of FN1, COL4A1, and LUM in the cells and in the supernatant mediums were elevated after RUNX1 overexpression in U-87 MG and N33 cells via performing western blot (WB) and enzyme linked immunosorbent assay (ELISA) assays (Fig. 2C–F). Meanwhile, we silenced RUNX1 via two lentiviruses carrying shRUNX1-1 or shRUNX1-3 in N9 and TBD0220 cells, and observed that silencing RUNX1 significantly downregulated both intracellular expression and extracellular paracrine of FN1, COL4A1, and LUM (Fig. 2G–L). Given that shRUNX1-3 targeted to the 3’ untranslated region of *RUNX1*, we rescued the expression level of RUNX1 by using lentivirus particle containing coding sequence of *RUNX1* in N9-shRUNX1-3 and TBD0220-shRUNX1-3 cells. Consistent with the previous results, the RUNX1 KD-induced downregulation of FN1, COL4A1, and LUM was reversed by ectopically expressing recombinant RUNX1 (Fig. 2G–L). These results suggested that RUNX1 regulated the expression and secretion of FN1, COL4A1, and LUM in GBM cells.

It is well-known that RUNX1 functions as a TF depending on its binding to the chaperone protein CBFβ and plays essential roles in a variety of cellular pathways. So, we sought to investigate whether RUNX1 might transcriptionally regulate the ECM-related genes in GBM. We performed siRNA-mediated CBFβ KD in N9 and TBD0220 cells and found that both the mRNA and protein levels of FN1, COL4A1, and LUM were not altered significantly between the groups (Supplementary Fig. S2A–D). We also utilized the SMI Ro5-3335 to block the interaction between RUNX1 and CBFβ at the chromatin level. WB and qRT-PCR results indicated this inhibitor had no significant decrease on the mRNA and protein levels of these genes (Supplementary Fig. S2E–H). These results thus indicated the role of RUNX1 in regulating the expression of FN1, COL4A1, and LUM via a TF-independent pathway in GBM.

By analyzing the ChIP-sequencing data, we found that RUNX1 was enriched at the promoter regions of *FN1*, *COL4A1*, and *LUM* genes, which was decreased in RUNX1-KD N9 cells. The level of histone H3K4me3 was also decreased in the promoter regions of these genes (Fig. 2M–O). To further verify the RUNX1 enrichment in the promoter regions of these genes, we employed ChIP-qRT-PCR assay by using an anti-RUNX1 antibody in N9 and TBD0220 cells. As shown in Fig. 2P and Supplementary Fig. S2I, RUNX1 was significantly enriched at the promoter regions of these genes, compared to the IgG control. However, RUNX1 KD in N9 and TBD0220 cells, H3K4me3 enrichment at the promoter regions of these genes was also significantly reduced (Fig. 2Q and Supplementary Fig. S2J).

Collectively, these results suggest that RUNX1 may promote the expression of FN1, COL4A1, and LUM factors via binding to their promoter regions and regulating the recruitment of H3K4me3 to the chromatin instead of acting directly as a TF.

### RUNX1 interacts with NPM1 regulating the expression of ECM-related genes

**Fig. 3:**
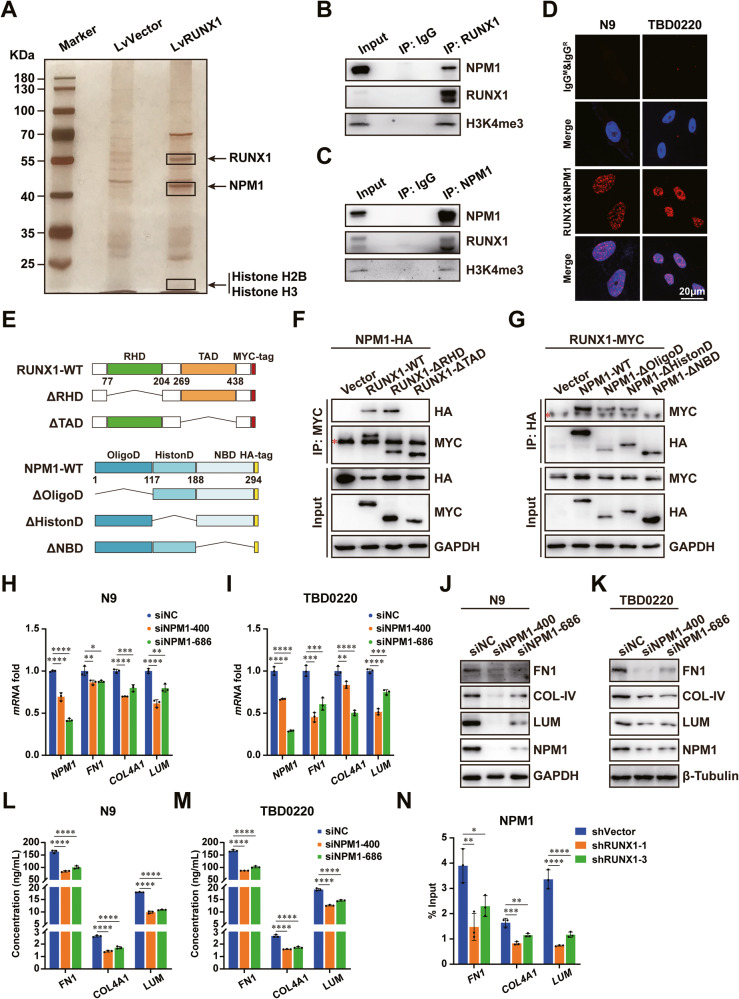
RUNX1 interacts with NPM1 for promoting the expression of ECM-associated genes. **A** Anti-FLAG antibody-pulled down samples from FLAG-RUNX1 expressing N9 cells were analyzed by the SDS-PAGE and silver staining. **B**, **C** Anti-RUNX1 or anti-NPM1 antibody co-IPed samples were analyzed by WB. **D** PLA was performed for detecting the interaction between RUNX1 and NPM1. **E** Schematic of various RUNX1 and NPM1 truncations. The domains annotated with residue numbers and truncations of RUNX1 and NPM1 were shown. RHD: runt-homology domain. TAD: transactivation domain. OligoD: oligomerization domain. HistonD: histone binding domain. NBD: nuclear acid binding domain. **F**, **G** Total cell lysates from HEK 293 T cells expressing different truncations of RUNX1 and NPM1 were IPed and immunoblotted with antibodies against HA or MYC tags. Red asterisk: heavy chain. The RUNX1 **H**, **I** The mRNA levels of *RUNX1*, *FN1*, *COL4A1*, and *LUM* in N9 and TBD0220 cells with NPM1 KD were detected by qRT-PCR assays. GAPDH served as the internal control. **J**, **K** The protein levels of RUNX1, FN1, COL4A1, and LUM in N9 and TBD0220 cells with NPM1 KD were detected by WB assays. GAPDH or β-Tubulin served as the internal control. **L**, **M** The levels of FN1, COL4A1, and LUM in the culture supernatants of N9 and TBD0220 cells transfected with siNPM1 were measured by ELISA. **N** ChIP results of the promoters of *FN1*, *COL4A1*, and *LUM* genes enriched by NPM1 in RUNX1 KD N9 cells. Student’s t-test for the two-group analysis, and one-way ANOVA for comparison of multiple groups. *P < 0.05, **P < 0.01, ***P < 0.001, ****P < 0.0001.

To understand how RUNX1 might regulate ECM-related genes’ expressions, N9 cells were stably infected with recombinant LvRUNX1 lentivirus for immuno-purification. Mass spectrometry (MS) analysis of RUNX1 interactome in N9-LvVector versus N9-LvRUNX1 cells identified NPM1, histone H2B, and H3 as the potential interacting partners in GBM cells (Fig. 3A and Supplementary Fig. S3A, B). The results of co-immunoprecipitation (co-IP) and proximity ligation assay (PLA) confirmed that RUNX1, NPM1, and H3K4me3 could physically interact with each other in GBM cells (Fig. 3B–D). By ectopically expressing different truncations of RUNX1 and NPM1, we performed co-IP assays to reveal that the transactivation domain (TAD) in RUNX1 and the nuclear acid binding domain (NBD) in NPM1 were vital for their interaction (Fig. 3E–G). Furthermore, siRNA-mediated depletion of NPM1 in N9 and TBD0220 cells significantly affected the binding efficiency of RUNX1 to H3K4me3 (Supplementary Fig. S3C, D). The interaction between NPM1 and H3K4me3 could be blocked in RUNX1-KD cells, which can be recovered by restoring RUNX1 expression (Supplementary Fig. S3E, F).

NPM1 acts as a chaperonin for histones H2B and H3, which are involved in various cellular processes such as ribosome biogenesis and histone assembly. To explore whether NPM1 might regulate the expression of ECM-related genes, we knocked down NPM1 and measured the expression of ECM-related genes in GBM cells by qRT-PCR and WB assays, which showed that expressions of FN1, COL4A1, and LUM were significantly reduced in NPM1 KD cells (Fig. 3H–K). Also, levels of these proteins were significantly decreased in the cell culture supernatants of NPM1-silenced GBM cells as measured by ELISA (Fig. 3L, M). Subsequently, we performed the ChIP-qRT-PCR assay to understand whether NPM1’s regulatory role on ECM-related genes might be controlled by RUNX1 expression in GBM cells. We found decreased enrichments of NPM1 on the promoter regions of these genes in shRUNX1-treated N9 and TBD0220 cell lines, compared to those treated with shVector (Fig. 3N and Supplementary Fig. S3G).

Together, these findings suggest that RUNX1 may function in association with NPM1 to regulate the expression of ECM-related genes in GBM cells.

### RUNX1-NPM1 interaction facilitates the chromatin accessibility of FOSL2 promoting the activation of ECM-related genes

**Fig. 4:**
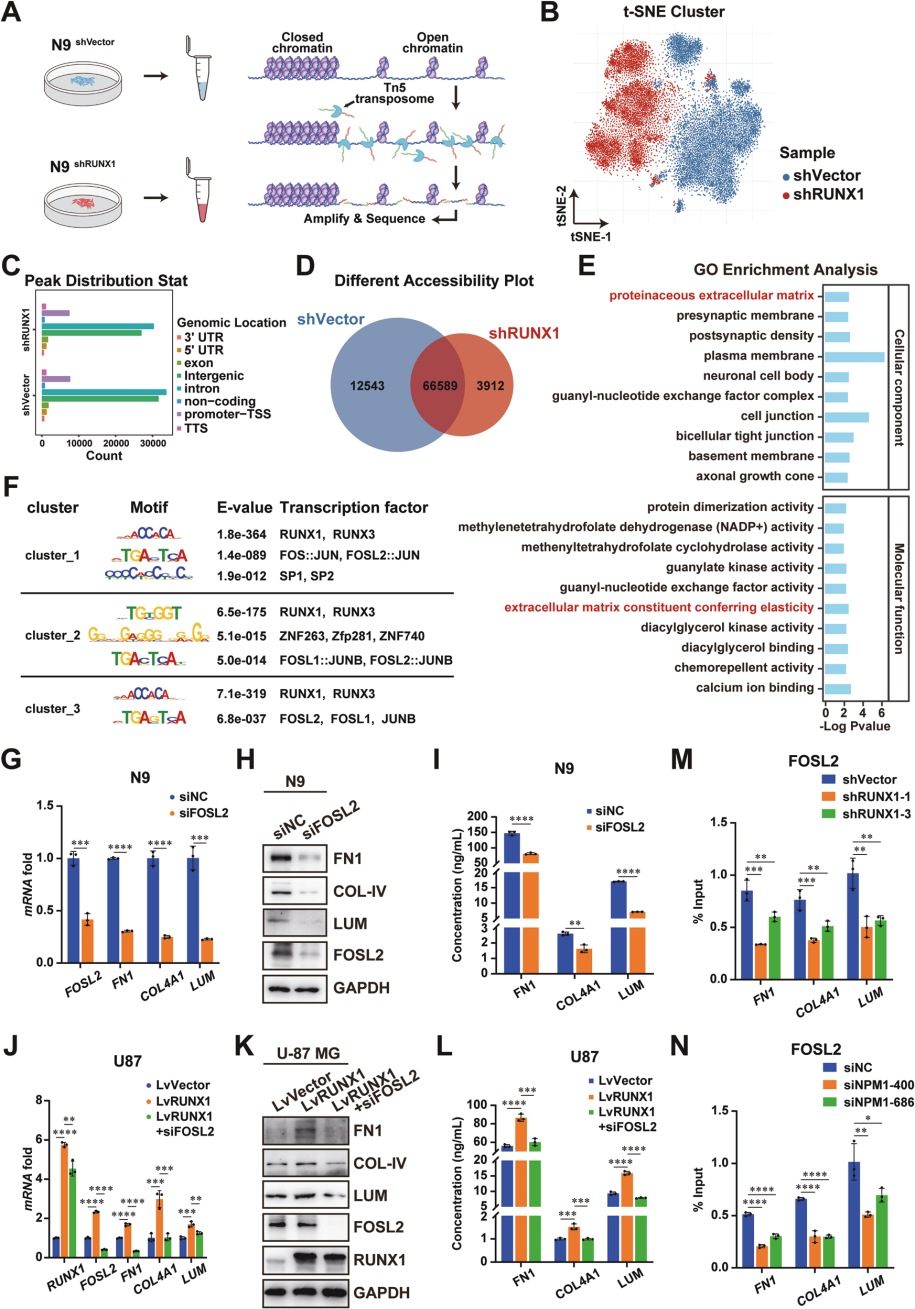
RUNX1 interacts with NPM1 to allow the chromatin accessibility and promote FOSL2-mediated expressions of ECM-related genes. **A** Schematic diagram of the single-cell ATAC-sequencing in N9 cells transfected with shVector or shRUNX1. **B** Clustering results of N9-shVector or shRUNX1 cells based on the ATAC-sequencing data were visualized as a tSNE plot. **C** Peak distribution analysis of RUNX1-associated chromatin regions in N9 cells transfected with shVector or shRUNX1. **D** Differential accessibility analysis in RUNX1 KD N9 cells was performed. **E** GO results of unique genes in N9-shVector cells. **F** Motif analysis and transcription factor prediction using RUNX1 ChIP-sequencing data. **G**–**I** The mRNA, protein, and secretion levels of FOSL2, FN1, COL4A1, and LUM were measured by qRT-PCR, WB and ELISA assays in N9 cells transfected with siNC or siFOSL2. GAPDH served as the internal control. **J**–**L** qRT-PCR, WB and ELISA results of the expression and secretion levels of RUNX1, FOSL2, FN1, COL4A1, and LUM in U-87 MG-LvVector, U-87 MG-LvRUNX1, or U-87 MG-LvRUNX1+siFOSL2 treated groups. GAPDH was used as the internal control. **M**, **N** ChIP-qRT-PCR results of FOSL2 occupying the promoter regions of ECM-related genes in N9 cells with RUNX1 or NPM1 KD. Student’s t-test for the two-group, and one-way ANOVA for comparisons of multiple groups. *P < 0.05, **P < 0.01, ***P < 0.001, ****P < 0.0001.

We uncovered that RUNX1 and NPM1 interaction could promote the expression of ECM-related genes. RUNX1 could increase the level of histone H3K4me3 in the promoter regions of ECM-related genes, suggesting that RUNX1 might be involved in the modulation of chromatin accessibility of the promoter regions of ECM-related genes. Therefore, we performed scATAC-sequencing analysis using the N9 cells transfected with shVector or shRUNX1 (Fig. 4A). After dimensional reduction and unsupervised clustering, the t-distributed Stochastic Neighbor Embedding (t-SNE) plot showed that RUNX1 KD could induce extensive changes in the chromatin landscape of N9 cells, compared with that in control cells (Fig. 4B). Although the peak distributions were not significantly altered between the N9 shVector and shRUNX1 groups (Fig. 4C), RUNX1 KD further showed reduced chromatin accessibility of 12543 genes in GBM cells (Fig. 4D). GO analysis of annotated genes with differential accessibilities in N9 shVector cells exhibited enrichment of ECM-associated functions (Fig. 4E).

To explore the RUNX1-dependent transcriptional regulation of ECM-related genes, we performed the motif enrichment analysis using the RUNX1 ChIP-seq data. The result in Fig. 4F showed that anti-RUNX1 antibody IPed sequences were sorted into 3 main clusters according to their different characteristic motifs. Each cluster had potentially interacting TFs, with FOSL2 being universally identified in all clusters. By analyzing available public GBM datasets from the Cancer Genome Atlas (TCGA) and the Chinese Glioma Genome Atlas (CGGA), we revealed that FOSL2 was highly expressed in mesenchymal GBM (Supplementary Fig. S4A–D), which was consistent with the expression characteristics of RUNX1. Silencing FOSL2 significantly decreased the mRNA, and protein levels of FN1, COL4A1, and LUM, as well as their extracellular secretion (Fig. 4G–I and Supplementary Fig. S4E–G). The upregulated levels of these genes by RUNX1 OE in U-87 MG and N33 cells were decreased after transfected with siFOSL2 (Fig. 4J–L and Supplementary Fig. S4H–J). Reduced levels of FOSL2 at the promoter regions of ECM-related genes were found in both N9 and TBD0220 cells with RUNX1 or NPM1 KD (Fig. 4M, N and Supplementary Fig. S4K, L). Likewise, RUNX1 OE showed pronounced enrichment of FOSL2 at the promoters of these genes in U-87 MG and N33 cells (Supplementary Fig. 4M, N). Therefore, these results suggest that RUNX1 may partner with NPM1 to facilitate the promoter-specific recruitment of FOSL2 for the transcriptional activation of ECM-related genes.

### RUNX1-induced increase in ECM-related genes expression confers poor prognosis and complex TIME in GBM

**Fig. 5:**
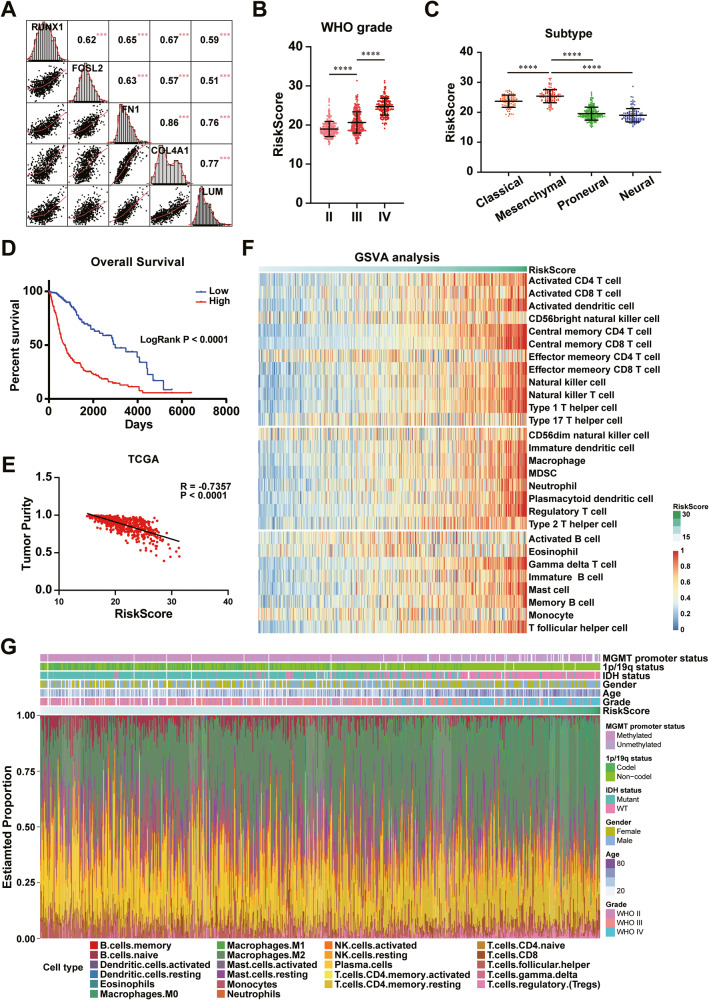
RUNX1, FOSL2, and ECM gene expression-based novel signature model correlating with tumor malignancy, survival time, and immunosuppressive microenvironment in GBM. **A** The correlation with the expression of RUNX1, FOSL2, FN1, COL4A1, and LUM was calculated by Spearman method in the TCGA GBM cohort. **B** Signature scores of different grades of gliomas in the TCGA GBM cohort were visualized as a scatter plot. **C** Scatter plot showing distribution of scores in the classical, mesenchymal, proneural, and neural subtypes of GBM in the TCGA GBM cohort. **D** Kaplan–Meier survival analysis was performed in the low-score and high-score groups. **E** The relationship between signature scores and tumor purity. **F** GSVA analysis of scores correlated with immune cell lineages in the TCGA GBM cohort. **G** CIBERSORTx analysis of scores correlated with infiltrated immune cell populations and distributions in the TCGA GBM cohort. One-way ANOVA for comparisons of multiple groups. ***P < 0.001, ****P < 0.0001.

Next, we interrogated the glioma cohorts from TCGA and CGGA databases to further examine the expression and correlation characteristics of RUNX1, FOSL2, FN1, COL4A1, and LUM in GBM tumorigenesis. The results of Spearman correlation analysis showed significant positive correlation among these genes (Fig. 5A and Supplementary Fig. S5A), which were consistent with our in vitro results. Thus, we attempted to integrate differential expressions of these genes for constructing a novel model for clustering and prognosis prediction of GBM patients. As shown in Fig. 5B and Supplementary Fig. 5B, a high signature score was positively correlated with high tumor grading or malignancy in this cohort analysis. Similar to the expression profiles of RUNX1 and FOSL2, higher signature scores were more frequently sorted in mesenchymal subtypes of GBM (Fig. 5C and Supplementary Fig. 5C). Patients with high scores presented shortened overall survival (OS) time, compared with those with low scores (Fig. 5D and Supplementary Fig. 5D).

ECM remodeling, which often occurs in advanced gliomas, orchestrates a series of malignant progressions, including tumor-infiltrated growth, metastasis, and immune escape, thereby not only providing physiological support to the tumor growth but also involving surrounding non-malignant cells through ligand-receptor interactions [18]. Therefore, we next investigated the relationship between signature scores and TIME in GBM. By in silico analysis, we found that the tumor purity was negatively correlated with the signature score (Fig. 5E and Supplementary Fig. 5E). GSVA analysis of infiltrated immune cells further revealed that signature scores had a significantly strong association with corresponding immune cell lineages (Fig. 5F and Supplementary Fig. 5F). Immune cell compositions of tumors were analyzed by the CIBERSORT algorithm. A high signature score was positively correlated with increased macrophage infiltration into the TIME (Fig. 5G and Supplementary Fig. 5G). Taken together, our results unveil a novel signature to predict the TIME status in GBM patients.

### RUNX1 promotes ECM remodeling and immunosuppressive microenvironment in GBM in vivo

**Fig. 6:**
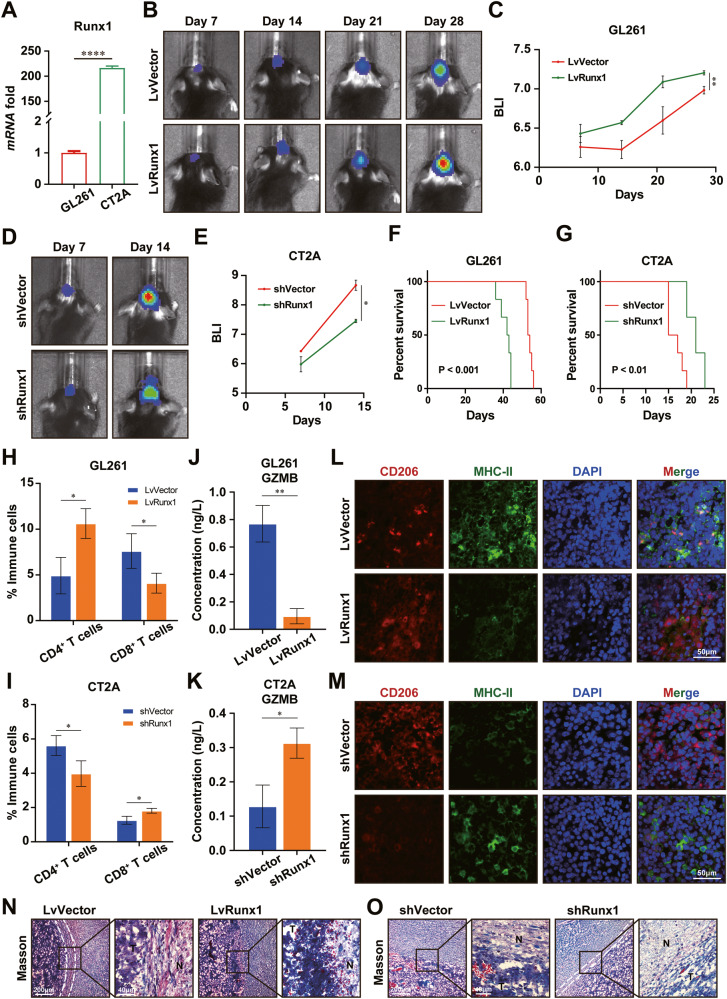
RUNX1-mediated ECM remodeling promotes tumor growth and immunosuppressive microenvironment in GBM. **A** qRT-PCR analysis of Runx1 expressions in GL261 and CT2A cells. **B**, **C** GL261 cells transfected with LvVector or LvRunx1 were intracranially injected to establish the orthotopic GBM model in C57BL6 mice (N = 6 per group). Bioluminescent imaging was performed on days 7, 14, 21, and 28 after injection. **D**, **E** Orthotopic GBM mice grafted with CT2A cells treated with shVector or shRunx1 were measured by bioluminescent imaging on days 7 and 14 after injection. N = 6 mice per group. Kaplan–Meier survival curve analysis for correlation between the Runx1 expression and survival time of GL261-grafted (**F**) and CT2A-grafted mice (**G**). Flow cytometry analysis of tumors infiltrated with CD4+ and CD8+ T cells in GL261 tumors (**H**) and CT2A tumors (**I**). ELISA analysis of GZMB levels in GL261 tumors (**J**) and CT2A tumors (**K**). Confocal imaging analysis of GL261 tumors (**L**) and CT2A tumors (**M**), showing the expression of M1 marker (MHC-II) and M2 marker (CD206) in macrophages. Brightfield images of Masson’s staining of GL261 tumors (**N**) and CT2A tumors (**O**), showing the fiber structures at the tumor margins. Student’s t-test for the two-group analysis. *P < 0.05, **P < 0.01, ****P < 0.0001.

To clarify the functional role of RUNX1 in the GBM malignancy in vivo, we established an orthotopic GBM model in immunocompetent C57BL/6 mice by using syngeneic cell lines GL261 and CT2A. Before constructing the model, we evaluated the expression of Runx1 in GL261 and CT2A cells, which showed a significantly higher expression level of Runx1 in CT2A, compared with that in GL261 (Fig. 6A). Therefore, we overexpressed recombinant Runx1 in GL261 cells and downregulated Runx1 expression in CT2A (Supplementary Fig. S6A, B). Tumor growth of tumor-bearing mice was monitored by animal bioluminescence imaging after intracranial injection of GL261 or CT2A cells. The results showed that Runx1 OE significantly promoted the tumor growth in vivo (Fig. 6B, C), while tumors in CT2A-shRunx1-implanted mice exhibited retarded growth rates (Fig. 6D, E), compared with the respective control mice. Kaplan-Meier curve analysis revealed that mice treated with GL261-LvVector or CT2A-shRunx1 had a longer OS time, compared with GL261-LvRunx1 or CT2A-shVector (Fig. 6F, G). Flow cytometry (FC) analysis of tumors revealed Runx1 OE accelerated infiltration of CD4^+^ T cells, in particular, while inhibiting the accumulation of CD8^+^ T cells in TIME. However, Runx1 KD decreased CD4^+^ T cells but increased CD8^+^ T cells infiltration into TIME (Fig. 6H, I). Tumors with high levels of Runx1 also had increased concentrations of GZMB as determined by ELISA (Fig. 6J, K). The increased population of CD206-positive M2-macrophages and decreased proportion of MHC-II-expressing M1-macrophages were observed in GL261-LvRunx1 or CT2A-shVector tumors than that in GL261-LvVector or CT2A-shRunx1 tumors (Fig. 6L, M). Subsequently, we performed Masson’s staining to evaluate the ECM status in GBM tumors, which indicated the presence of a border band (white line) between borders of low levels of Runx1 expressing tumors, whereas the border band at the tumor-normal tissue junction disappeared in Runx1 OE tumors, implying that the ECM remodeling might be induced by Runx1 in GBM tumors (Fig. 6N, O). Taken together, these results demonstrate the vital role of RUNX1 in the ECM remodeling and maintaining immunosuppressive microenvironment in GBM.

## Discussion

RUNX1, well-characterized for its essential and crucial roles in the hematopoietic stem cells, has recently been found to play pivotal roles in GBM malignancy. Through an integrated analysis of gene expression profiles of 76 GBM patients, Carro MS. et al identified six key TFs, including RUNX1, that might contribute to the mesenchymal subtype of GBM [15]. Furthermore, by comparing the gene expression profiles and miRNA expression profiles of 206 GBM patients by bioinformatics computing, Sumazin et al. have detected six key genes, including RUNX1, that could significantly regulate GBM tumorigenesis and help reveal the mesenchymal subtype [16]. Previously, we conducted a multi-dimensional integral analysis of 560 primary GBM patients’ RNA microarray, miRNA, and STRING protein network databases. Based on the competitive endogenous RNA (ceRNA) regulatory network analysis, we identified core regulatory factors, including RUNX1, that might facilitate the maintenance of the mesenchymal subtype of GBM [19]. RUNX1 has been subsequently found to promote GBM cell proliferation and invasion in a TGFβ signaling-dependent manner [17]. Different studies have confirmed the close relationship between RUNX1 levels and mesenchymal GBMs. In this study, we found that the chromatin-binding region of RUNX1 was enriched in the promoter regions of ECM-associated gene in GBM cells, and then revealed that alterations in the expression level of RUNX1 might regulate the downstream expressions and secretions of FN1, COL4A1, and LUM. In vivo experiments showed that high RUNX1 expression levels could induce ECM remodeling and immunosuppressive microenvironment in GBM, and vice versa. These results suggest that targeting RUNX1 might have a strong therapeutic potential in GBM.

RUNX1 mutations, including frameshifts, missense mutations, and gene fusions, are found in a wide variety of tumors such as AML, as well as RUNX1 SNPs in lung adenocarcinoma and colorectal cancer [20, 21]. However, RUNX1 has not been shown to carry any pathogenic mutation effects on gliomas [17], suggesting its distinctive critical roles in gliomas. We employed multi-omics approaches combined with in vitro experiments to uncover a novel regulatory mechanism of RUNX1 in GBM, demonstrating that RUNX1 interacted with NPM1 to promote chromatin accessibility and H3K4me3 modification, enhancing FOSL2-mediated transcriptional activation of ECM-related genes, ultimately leading to the ECM remodeling in GBM.

High spatial and temporal heterogeneity are distinct pathological hallmarks of GBM, which can also act as the driving force for developing chemotherapy resistance and tumor recurrences [22]. Based on the molecular expression characteristics, GBM can be classified into four molecular subtypes, namely classical, mesenchymal, proneural, and neural subtypes [23]. Subsequent reports suggest that the neural subtype should be culled [24]. Among those subtypes, the mesenchymal subtype is recognized as the major GBM subtype with the greatest heterogeneity and the most complex TIME. Recurrent gliomas also transition to the mesenchymal subtypes [25]. In silico analysis showed that RUNX1 and FOSL2 were significantly enriched in the mesenchymal GBM (Cell Death Dis, 2019 [17] and Supplementary Fig. S4C, D). The prognostic signature based on the expression profiles of RUNX1, FOSL2, and other ECM-related genes could be effectively utilized in predicting the prognosis and OS of glioma patients. High scores invariably showed relatively shorter OS. Signature scores were significantly associated with the extent of immunosuppression in the glioma microenvironment. Both in vitro and in vivo results confirmed that RUNX1-mediated ECM remodeling promoted pathologically increased infiltration of M2 macrophages and decreased the number of cytotoxic T cells, which finally formed an immunosuppressive microenvironment in GBM.

Our study identified FOSL2 as an important TF that might play roles in activating ECM gene transcription in a RUNX1-dependent manner. FOSL2 is known as a member of the activator protein-1 (AP-1) family, which plays important roles in multiple aspects of tumor development [26]. FOSL2 can not only maintain tumor inflammation and lead to tumor metastasis through the SOX2-FOSL2-IL6 axis but also promote M2 polarization of tumor-associated macrophages in a β-catenin-dependent manner [27, 28]. In gliomas, FOSL2 promotes tumors’ natural evolution and bone-marrow-derived macrophage polarization through the FOSL2-ANXA1-FPR1/3 axis in response to hypoxia signaling [29]. Our study demonstrated that FOSL2 transcriptionally activated the expression levels of ECM genes to induce ECM remodeling and increase M2 macrophage infiltration in the GBM TIME. Moreover, M2 macrophages can secrete TGFβ1, which could further activate the TGFβ-SMAD3 signaling pathway, promoting RUNX1 translocation into the nucleus. Besides, an enhanced RUNX1 expression was observed under hypoxic conditions (data not shown). Taken together, we hypothesized that an increased FOSL2 level in response to hypoxia could elevate the RUNX1 expression, mediating ECM remodeling in GBM. Also, FOSL2-induced infiltration of M2 macrophages into the TIME and ECM remodeling can further promote RUNX1’s nuclear translocation, thus forming a positive feedback loop during the GBM progression.

In conclusion, our findings uncovered the functional role of RUNX1 in GBM pathogenesis and progression. Mechanistically, RUNX1 promoted the chromatin accessibility and H3K4me3 modification via its interaction with NPM1 to enhance the transcriptional activation of FOSL2 towards ECM-related genes, resulting in the ECM remodeling and development of immunosuppressive microenvironment in GBM. Inhibiting RUNX1-mediated ECM remodeling might be a potent druggable target for reversing the “cold tumor” state of GBM and enhancing the antitumor effects of immunotherapies on GBM.

## Materials and methods

### Cell culture, lentivirus, plasmids, and chemicals

Human GBM cell line U-87MG, mouse GBM cell lines GL261, and human non-malignant cell line HEK 293T were purchased from the American Type Culture Collection (ATCC). The mouse GBM cell line CT2A was obtained from BLUEFBIO Co. Ltd. (#BFN60810497, Shanghai, China). The human primary GBM cell N9 and N33 were generously gifted from Professor Xiaolong Fan at Beijing Normal University. The human primary GBM cell TBD0220 was constructed by our laboratory. U-87MG, GL261, CT2A, and HEK 293T were cultured in Dulbecco’s Modified Eagle Medium (DMEM) supplemented with 10% fetal bovine serum (FBS). N9, N33, and TBD0220 were cultured in DMEM/F12 medium supplemented with 10% FBS. Cells were grown at 37°C in a humidified incubator with 5% CO_2_. Lentiviral vectors encoding human RUNX1 or mouse Runx1 were purchased from Genechem Corporation (Shanghai, China). Lentiviral vectors carrying short-hairpin RNA against RUNX1 (shRUNX1) or Runx1 (shRunx1) and siRNA targeting FOSL2 mRNA were obtained from IBS Biotech. Co., Ltd (Shanghai, China). Also, siRNAs targeting NPM1 and CBFβ were synthesized by GenePharma Corporation (Shanghai, China). Recombinant plasmids with wild type or different truncations of RUNX1 and NPM1 were constructed by IBS Biotech. Co. Ltd. For stable cell line constructions, cells were transduced with lentiviral particles and screened with 2 μg/mL of puromycin (#P8230, SolarBio Science & Technology Co., Ltd., Beijing, China). For transient transfection of siRNAs and plasmids, cells were seeded in culture plates and transfected with Lipofectamine-3000 reagent (#L3000015, Thermo Fisher, Waltham, MA, USA). The shRNA and siRNA sequences are presented in Supplementary Table S1. Ro5-3335 (#T4687) was purchased from TargetMol Corporation (Wellesley, MA, USA).

### Chromatin immunoprecipitation (ChIP)

Cells were cross-linked by 1% formaldehyde (#F8775, Sigma-Aldrich, St. Louis, MO, USA), followed by harvesting, sonication for chromatin fragmentation, and preparation of nuclear lysates. Then, lysates were incubated with the target primary antibody along with ChIP-grade magnetic beads (#16-663, Millipore, Billerica, MA, USA) overnight at 4 °C. After washing, reversing the cross-link, and purifying the eluate, ChIPed samples were subjected to the library preparation for sequencing or used to measure gene expressions by quantitative polymerase chain reaction (qPCR) in a thermocycler instrument (ABI QuantStudio 3 Real-time PCR System). ChIP primer sequences are summarized in Supplementary Table S2.

### Single-cell assay for transposase-accessible chromatin with high-throughput sequencing (scATAC-sequencing)

The adherent cells were digested by trypsin to the single cell suspensions, following by centrifugation to remove the supernatant. The cells were washed twice by wash buffer (PBS containing 0.04% BSA). Then added chilled lysis buffer [Tris-HCl (pH 7.4): 10 mM, NaCl: 10 mM, MgCl_2_: 3 mM, Tween-20: 0.1%, Nonidet P40: 0.1%, Digitonin: 0.01%, BSA: 1%, dissolved in the nuclease-free water] to resuspend and incubate for 5 min on ice. Pellet the cell nuclei at 500 rcf for 5 min at 4 °C. Resuspended the cell nuclei using chilled wash buffer [Tris-HCl (pH 7.4): 10 mM, NaCl: 10 mM, MgCl_2_: 3 mM, BSA: 1%, Tween-20: 0.1%, dissolved in the nuclease-free water] and centrifuged at 500 rcf for 5 min at 4 °C. The cell nuclei pellets were resuspended by chilled diluted nuclei buffer (#2000153/2000207, 10× Genomics, San Francisco, CA, USA). The cell nuclei concentration was determined by using a Countess II FL Automated Cell Counter. Then the nuclei suspensions were immediately used for library construction.

The library construction was performed according to the user guide of Chromium Single Cell ATAC Reagent Kits (v1.1 Chemistry). The nuclei suspension was mixed with ATAC buffer (#2000122, 10 × Genomics, San Francisco, CA, USA) and ATAC enzyme (#2000123/2000138, 10 × Genomics, San Francisco, CA, USA), following by incubating for 60 min at 37 °C in a thermal cycler for transposition. During the PCR reaction, prepared the Master Mix on ice containing Barcoding Reagent B (#2000194, 10 × Genomics, San Francisco, CA, USA), Reducing Agent B (#2000087, 10 × Genomics, San Francisco, CA, USA), and Barcoding Enzyme (#2000125/2000139, 10 × Genomics, San Francisco, CA, USA) on ice, pipetted mix and centrifuged briefly. After finishing Chip assembly, added Master Mix and transposed nuclei to the chip. The Single Cell ATAC Gel Beads (#2000210, 10 × Genomics, San Francisco, CA, USA) and Partitioning Oil (2000190, 10 × Genomics, San Francisco, CA, USA) were loaded onto the chip. Then closed the lid and placed the assembled chip with gasket in the tray for Gel Beads-in-emulsion (GEM) generation in a Chromium Controller. After finished, transferred GEMs into a new tube and performed a PCR reaction in a thermal cycler with the following protocol: 72 °C for 5 min, 98 °C for 30 s, 98 °C for 10 s, 59 °C for 30 s, 72 °C for 1 min, go to step 3 for a total of 12 cycles, and finally 15 °C hold. After post GEM incubation cleanup with Dynabeads and SPRIselect, the samples were used for next library construction. Sample Index PCR MIX containing Amp Mix (#2000047/2000103, 10 × Genomics, San Francisco, CA, USA) and SI-PCR Primer B (#2000128, 10 × Genomics, San Francisco, CA, USA) and an individual Single Index N Set A (#3000427, 10 × Genomics, San Francisco, CA, USA) were mixed with each sample and incubated in a thermal cycler with the following protocol: 98 °C for 45 s, 98 °C for 20 s, 67 °C for 30 s, 72 °C for 20 s, go to step 2 for a total of 11 cycles, and finally 4 °C hold. After post sample index double sided size selection by SPRIselect, library construction QC and quantification, the libraries were sequenced by Illumina sequencer.

### Western blotting (WB)

Cell lysates were harvested using RIPA lysis buffer (#R0010, Solarbio) with 1x protease inhibitor cocktail (PIC) (#HY-K0010, MCE) and denaturized at 99°C for 10 min. An equal amount of protein samples were separated by the SDS-PAGE and transferred onto polyvinylidene difluoride (PVDF) membranes. After blocked with 5% skim milk at room temperature for 2 h, PVDF membranes were incubated with targeted primary antibodies at 4 °C overnight. Then PVDF membranes were washed 3 times using PBST buffer (0.1% Tween-20 in PBS) and incubated with secondary antibodies at room temperature for 1 h. After rewashed 3 times using PBST buffer, the chemiluminescence detection method was adopted to image protein bands using the ProteinSimple apparatus. Antibody details are summarized in Supplementary Table S3.

### Quantitative real-time polymerase chain reaction (qRT-PCR)

Total RNA was extracted from each sample using the TRIzol reagent (#15596-026, Thermo Scientific), following the manufacturer’s instructions, and an equal amount of total RNA was used from each sample to synthesize the complementary DNA (cDNA) using reverse transcriptase kit (#K1622, Thermo Scientific). Briefly, 5 μg total RNA was incubated with 1 μL oligo(dT) primer, 4 μL reaction buffer, 2 μL dNTP mix, 1 μL RNase inhibitor, and 1 μL reverse transcriptase in a PCR tube, and at 42 °C for 60 min. Then, qRT-PCR assay was performed using SYBR Green mix (#Q711, Vazyme) on QuantStudio 3 Real-Time PCR system (Thermo Scientific). The primer sequences are presented in Supplementary Table S4.

### RNA sequencing (RNA seq)

Total RNAs were isolated from N9-shRUNX1 or N9-shVector cells by TRIzol reagent according to the manufacturer’s protocol. The cDNA libraries were constructed and sequenced by the Illumina HiSeq4000 platform. Hisat2 tool was used for aligning sequencing data to human reference genome (hg19). Differentially expressed genes were obtained by using DESeq2 R package.

### Enzyme-linked immunosorbent assay (ELISA)

Cell culture supernatants were collected from all groups and centrifuged at 1000 × *g* for 10 min at 4 °C. Then the supernatants were used for target protein concentration determination according to the manufacturer’s instructions. Standards were prepared, according to the manufacturer’s instructions. Samples or standards were added to coated plates and incubated at 37 °C. The plates were subsequently washed five times with a wash solution. The supernatants were discarded, the enzyme-labeled reagents were added, and the plates were incubated again at 37 °C. After 5 times of washing and HRP reaction, the plates were read at OD450 in a microplate reader, and the sample concentrations were calculated from the standard curve. The ELISA kit for human FN1 (#LP-H06200) and COL4A1 (#LP-H04479) were purchased from Shanghai Lanpai Biotechnology. The human LUM ELISA kit (#E-EL-H0198c) was purchased from Elabscience. And the mouse GZMB ELISA kit (MM-0339M1) was purchased from Jiangsu Meimian Industrial Co., Ltd.

### Co-immunoprecipitation (Co-IP) assay

Cells were washed twice and collected with ice-cold PBS into microcentrifuge tubes. After centrifuge, cell pellets were dissociated by NP-40-containing lysis buffer (#P0013F, Beyotime) with 1 × PIC. Then samples were incubated on ice for 15 min. After centrifuging for 10 min at 4 °C, total cell lysates were prepared. For precleaning, cell lysates were incubated with 20 μL magnetic beads for 2 h at 4°C in a rotor. Then, magnetic beads were removed using a magnetic separation rack. An aliquot of 1 mg of total protein was mixed with 20 μL magnetic beads and appropriate dilution of antibodies, as recommended in the product datasheet, and kept overnight at 4 °C in a rotor. After washing 5 times with IP wash buffer, pellets were resuspended with 2 × protein loading buffer and denatured for 10 min at 99 °C. Samples were separated by running in the SDS-PAGE. For the mass spectrometry (MS) analysis, a silver staining kit (#24612, Thermo Scientific) was used following the manufacturer’s instructions. Bands were excised from the gel based on the silver staining. After dissolution and enzymatic digestion, samples were detected by a mass spectrometer. For WB analysis, the gels were transferred onto PVDF membranes for incubation with antibodies.

### Proximity ligation assay (PLA)

The PLA assay was performed according to the manufacturer’s protocol. Briefly, cells were seeded into 96-well plates with glass slides, fixed by 4% paraformaldehyde at room temperature for 10 min, and permeabilized by 0.2% Triton-X 100 at room temperature for 10 min. After washed twice by PBS buffer, the slides were incubated with blocking solution at 37 °C for 60 min, followed by incubated with two primary antibodies at 4 °C overnight. Then, the slides were washed twice by Wash Buffer A (#DUO82049, Sigma) at room temperature and incubated with the PLA plus (#DUO92002, Sigma) and minus probes (#DUO92004, Sigma) for 1 h at 37 °C in a humidified chamber. After incubation, the slides were subjected to wash twice by Wash Buffer A and incubation with ligation mix (#DUO92008, Sigma) at 37 °C for 30 min. Then the slides were washed twice by Wash Buffer A and incubated with amplification mix at 37 °C for 100 min. After the final washes of Wash Buffer A twice and Wash Buffer B (#DUO82049, Sigma) once, the slides were mounted with DAPI-containing mounting medium (#F6057, Sigma) and imaged under a confocal microscope (Olympus).

### Orthotopic GBM model construction

All animal experiments were approved by the Animal Ethical and Welfare Committee of Tianjin Medical University (TMU). The female C57BL/6J mice were purchased from the Beijing Vital River Laboratory Animal Technology Company Limited and housed in a specific pathogen free (SPF) grade condition. All the mice were randomized before modeling. The orthotopic GBM model was constructed as described elsewhere [30]. Briefly, GL261 or CT2A cells were intracranially injected into the mice. Bioluminescence imaging after performing intraperitoneal injection of beetle luciferin (#E1605, Promega) was performed to measure the tumor growth by In Vivo Imaging System (IVIS) Spectrum.

### Flow cytometry (FC) analysis of tumor-infiltrated T cells

GBM tumors were digested to single cell suspensions and stained with anti-CD45.2, anti-CD3, anti-CD4, and anti-CD8 antibodies, according to the manufacturer’s protocol. Briefly, tumor-bearing mice were sacrificed, and tumors were harvested by surgical resections. Then, tumors were cut into very small pieces by scissors and digested using collagenase and DNaseI to prepare the single-cell suspension. Red blood cells (RBCs) were removed using an RBC lysis buffer (#R1010, Solarbio). After being filtered through 70μm cell strainers, samples were blocked by anti-CD16/32 antibodies (#101319, BioLegend). T cells were stained with anti-CD45.2 (#109806, BioLegend), anti-CD3 (#100236, BioLegend), anti-CD4 (#100408, BioLegend), and anti-CD8 (#100734, BioLegend) antibodies, according to the manufacturer’s protocols. After washing off residual antibodies, samples were analyzed on the BD FACSVerse instrument. CD4^+^ T cells were defined as CD45.2^+^, CD3^+^, CD4^+^, and CD8^-^; CD8^+^ T cells were defined as CD45.2^+^, CD3^+^, CD4^−^, and CD8^+^. Results were analyzed by FlowJo v10.6.2 software.

### Immunofluorescence (IF) analysis of macrophage-infiltration in tumor tissues

Tumor tissues were fixed by formalin and embedded using the Tissue-Tek O.C.T compound (#4583, Sakura). Samples were sectioned by a Cryostats apparatus (Leica). After washing with PBS buffer, and blocking with block solution, tissue sections were incubated with anti-CD206 (#24595S, CST) and anti-MHC-II (#ab23990, Abcam) antibodies overnight at 4 °C. The next day, samples were washed 3 times with PBS buffer and incubated with fluorescent secondary antibodies, followed by mounting with a DAPI-mounting medium (#F6057, Sigma). Images were captured by a confocal microscope (Olympus).

### Masson’s trichrome staining

Frozen sections were employed to perform Masson’s staining using a Masson’s trichrome staining kit (#G1340, Solarbio), according to the manufacturer’s instructions. Briefly, prefixed samples were incubated overnight with Bouin’s solution, followed by Weigert′s iron hematoxylin staining, ponceau-fuchsin counterstaining, and phosphomolybdic acid staining. Then samples were sealed using neutral balsam and imaged by bright microscopy (Olympus).

### Statistical analysis

All statistical analyses were performed using GraphPad Prism 8 software. Student’s t-test was used for comparing the two groups, and one-way or two-way analysis of variance (ANOVA) for comparing multiple groups. The error bars represent mean ± standard deviation (SD) with extra data points. Statistical significance was considered at *P < 0.05, **P < 0.01, ***P < 0.001, ****P < 0.0001, or *P > 0.05.

### Reporting summary

Further information on research design is available in the Nature Research Reporting Summary linked to this article.

### Supplementary information

Supplementary Materials

Reporting Summary

## Data Availability

All sequencing and metabolomics data in this study are available from the corresponding author upon reasonable request.
