# Supplementary material for: RUNX1/NPM1/H3K4me3 complex contributes to extracellular matrix remodeling via enhancing FOSL2 transcriptional activation in glioblastoma
Source: Cell Death Dis. 2024 Jan 29;15(1):98. doi: 10.1038/s41419-024-06481-4 (PMC10825180; doi:10.1038/s41419-024-06481-4)

Supplementary Materials

RUNX1/NPM1/H3K4me3 complex contributes to extracellular matrix remodeling via enhancing FOSL2 transcriptional activation in glioblastoma

Xiaoteng Cui, Dawei Huo, Qixue Wang, Yunfei Wang, Xiaomin Liu, Kai Zhao Yongping You, Junxia Zhang*, Chunsheng Kang*

*** Corresponding author:**
Chunsheng Kang, Email: kang97061@tmu.edu.cn
Junxia Zhang, Email: zjx232@njmu.edu.cn

This PDF file includes:

***Supplementary* Figure S1-6**

***Supplementary* Table S1-5**

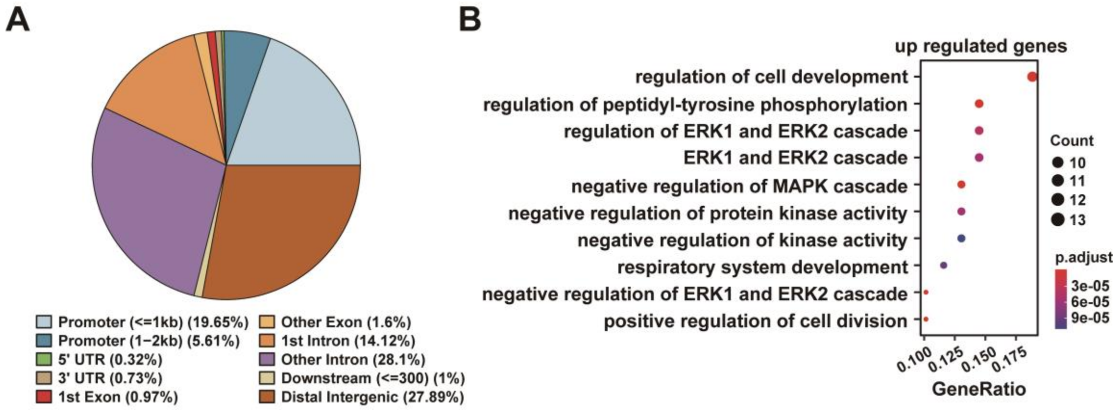

Supplementary Figure 1

***Supplementary* Fig. S1, related to Fig. 1.** (**A**) A Pie chart of annotated peak distributions enriched by RUNX1 in N9-shVector cells. (**B**) Gene Ontology (GO) analysis of 79 upregulated genes.

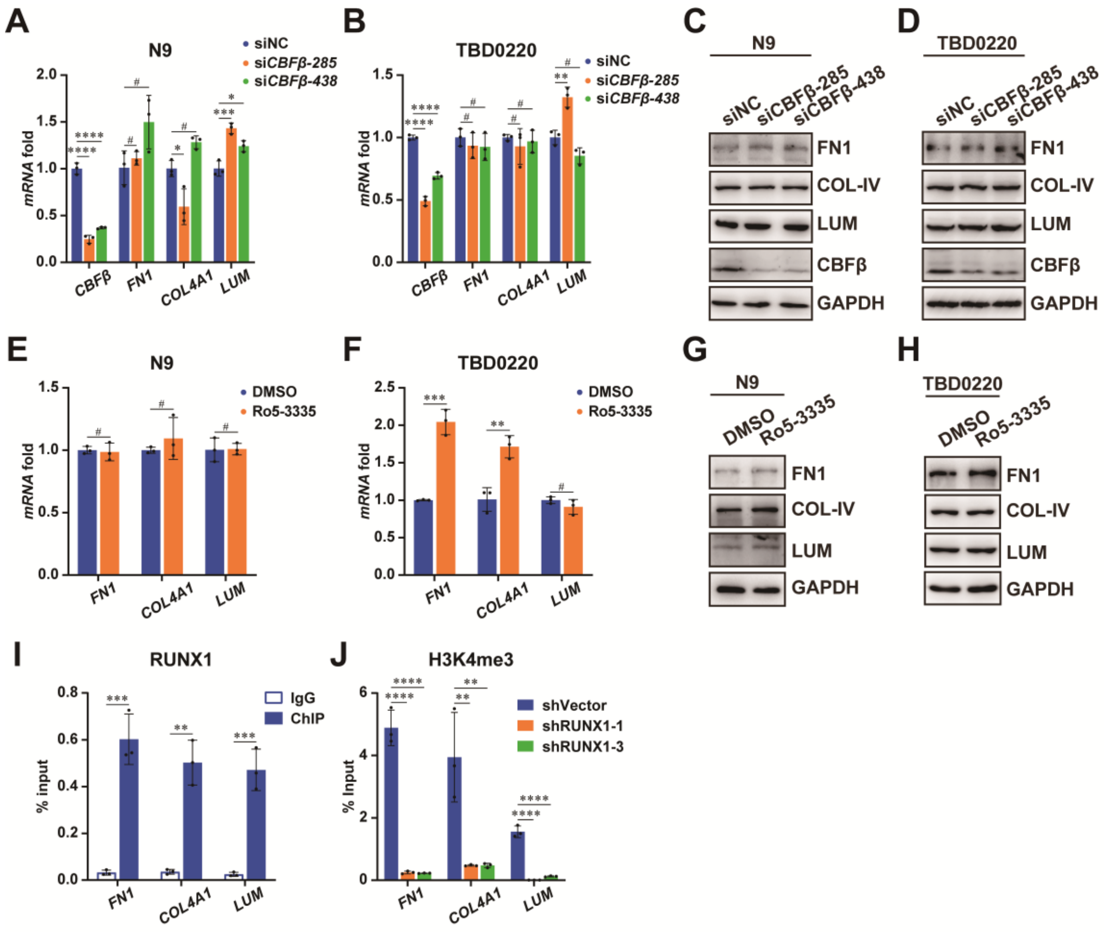

Supplementary **Figure 2**

Supplementary **Fig. S2, related to Fig. 2.** (**A-B**) The mRNA levels of *CBFβ*, *FN1*, *COL4A1*, and *LUM* in N9 and TBD0220 cells with RUNX1 KD and restored expression were respectively detected by qRT-PCR analyses. *GAPDH* was used as the internal control. (**C-D**) The protein levels of CBFβ, FN1, COL4A1, and LUM in N9 and TBD0220 cells with RUNX1 KD and restored expression were respectively detected by WB analyses. GAPDH was used as the internal control. (**E-H**) The mRNA and protein levels of FN1, COL-IV, and LUM isolated from N9 and TBD0220 cells treated with a vehicle (DMSO) or Ro5-3335 were respectively detected by qRT-PCR and WB assays. (**I**) ChIP analysis of the promoter regions of *FN1*, *COL-IV*, and *LUM* genes occupied by RUNX1 in TBD0220 cells. (**J**) H3K4me3 modifications in the promoter regions of *FN1*, *COL-IV*, and *LUM* were detected by ChIP-qPCR assays after downregulating RUNX1 in TBD0220 cells. Student's t-test was performed for the two-group analysis, and one-way ANOVA for comparisons of multiple groups. $^{\#}P \geq 0.05$, $^{*}P < 0.05$, $^{**}P < 0.01$, $^{***}P < 0.001$, $^{****}P < 0.0001$.

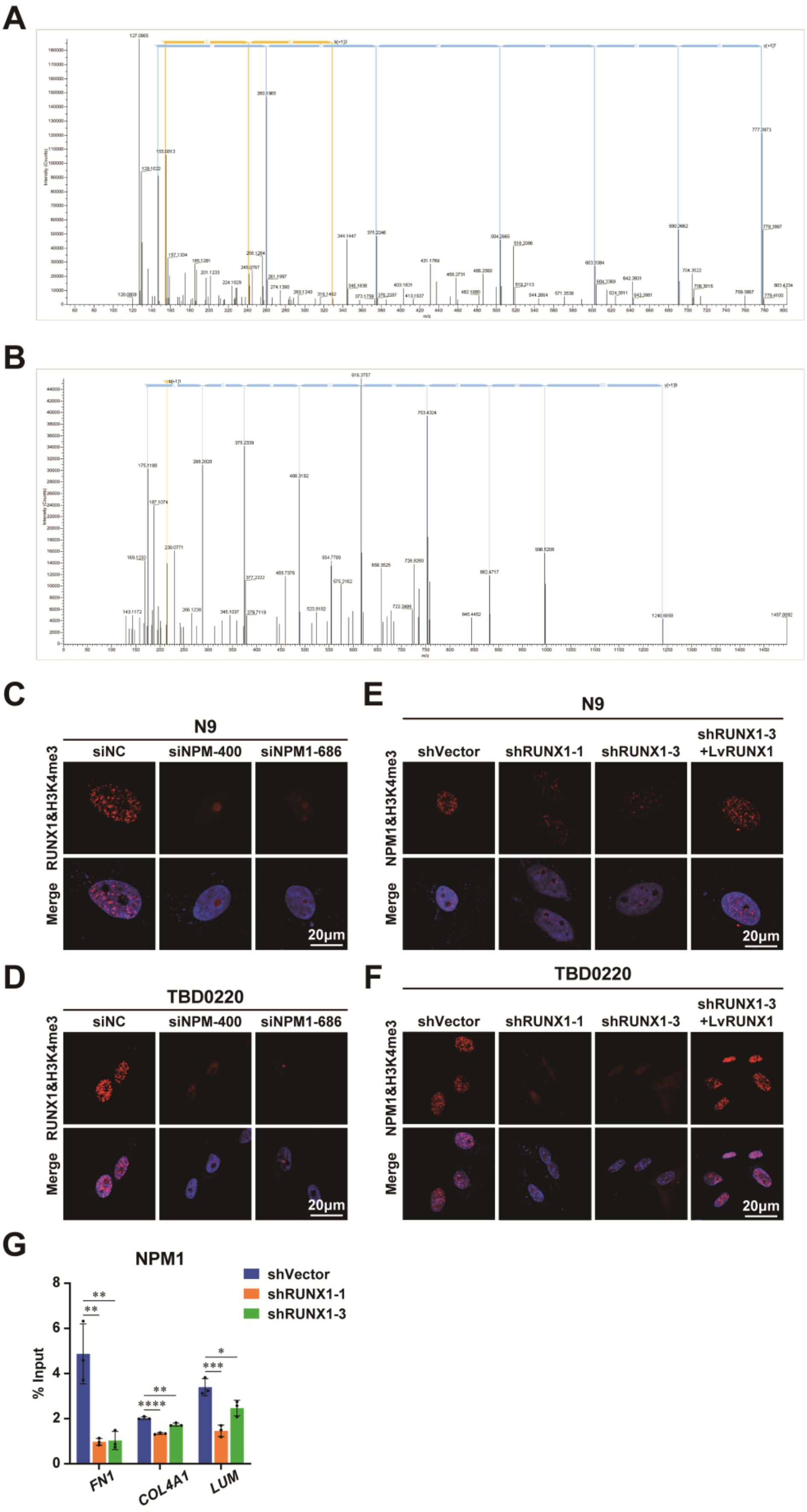

***Supplementary* Figure 3**

***Supplementary* Fig. S3, related to Fig. 3.** (**A-B**) Base peak chromatogram of NPM1, enriched by RUNX1. (**C-D**) The interaction between RUNX1 and H3K4me3 was measured by PLA assay in N9 and TBD0220 cells transfected with siNPM1. (**E-F**) PLA results of co-localization between NPM1 and H3K4me3 in N9 and TBD0220 cells with RUNX1 KD or restored expression. (**G**) ChIP analysis of the promoter regions of *FN1*, *COL-IV*, and *LUM* genes enriched by NPM1 in RUNX1-downregulated TBD0220 cells. One-way ANOVA was used for comparisons of multiple groups. $^{*}P < 0.05$, $^{**}P < 0.01$, $^{***}P < 0.001$, $^{****}P < 0.0001$.

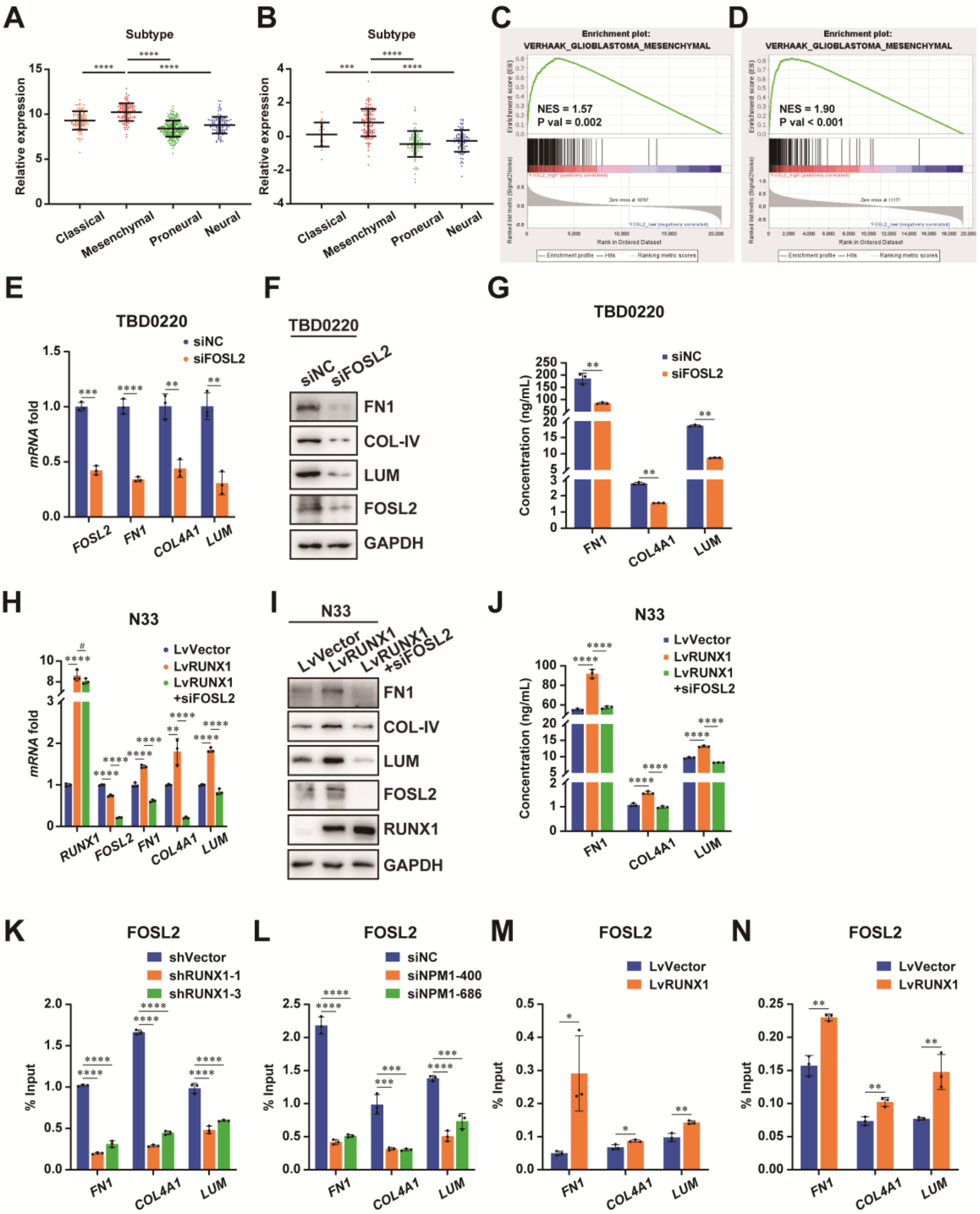

***Supplementary* Figure 4**

***Supplementary* Fig. S4, related to Fig. 4.** (**A-B**) Scatter plot showing the distribution of the FOSL2 expression in the classical, mesenchymal, proneural, and neural subtypes of GBM in TCGA (**A**) and CGGA (**B**) GBM cohorts. (**C-D**) GSEA analysis of FOSL2 expressions in TCGA (**C**) and CGGA (**D**) GBM cohorts. (**E-G**) The mRNA, protein, and secretion levels of FOSL2, FN1, COL4A1, and LUM were measured by qRT-PCR, WB and ELISA assays in TBD0220 cells transfected with siNC or siFOSL2. GAPDH

served as the internal control. (**H-J**) qRT-PCR, WB and ELISA results of the expression and secretion levels of RUNX1, FOSL2, FN1, COL4A1, and LUM in N33-LvVector, N33-LvRUNX1, or N33-LvRUNX1+siFOSL2 treated groups. GAPDH was used as the internal control. (**K-L**) ChIP-qPCR results of FOSL2 occupying the promoter region of ECM-associated genes in RUNX1 or NPM1 downregulated TBD0220 cells. (**M-N**) ChIP-qPCR results of FOSL2 occupying the promoter of ECM-associated genes in U-87 MG (**M**) and N33 cells (**N**) with or without RUNX1 overexpression. Student's t-test was applied for the two-group analysis, and one-way ANOVA for comparisons of multiple groups. $^{\#}P \geq 0.05$, $^{*}P < 0.05$, $^{**}P < 0.01$, $^{***}P < 0.001$, $^{****}P < 0.0001$.

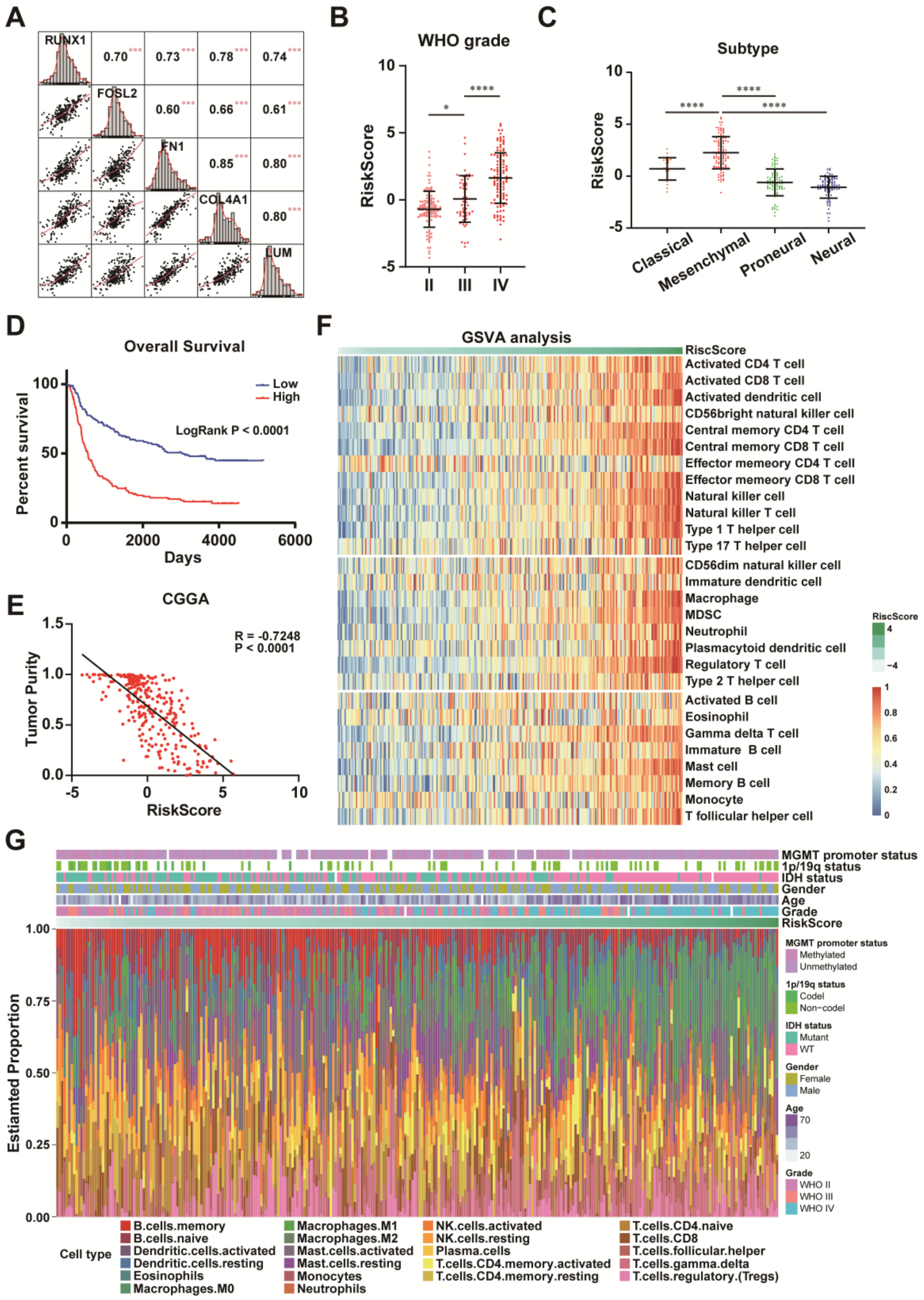

Supplementary Figure 5

***Supplementary* Fig. S5, related to Fig. 5.** (**A**) Correlations among expressions of RUNX1, FOSL2, FN1, COL4A1, and LUM were assessed by the Spearman method in the CGGA GBM cohort. (**B**) Signature scores of different tumor grades in the CGGA

GBM cohort were visualized as a scatter plot. (**C**) Scatter plot showing the distribution of scores in the classical, mesenchymal, proneural, and neural subtypes of GBM in the CGGA GBM cohort. (**D**) The Kaplan-Meier curve was analyzed for evaluating survival in the low-score and high-score groups. (**E**) The relationship between signature scores and tumor purity. (**F**) GSVA analysis of scores was correlated with corresponding immune cell lineages in the CGGA GBM cohort. (**G**) CIBERSORTx analysis of scores correlated with infiltrated immune cell populations and their distributions in the CGGA GBM cohort. One-way ANOVA for comparisons of multiple groups. $^{***}P < 0.001$, $^{****}P < 0.0001$.

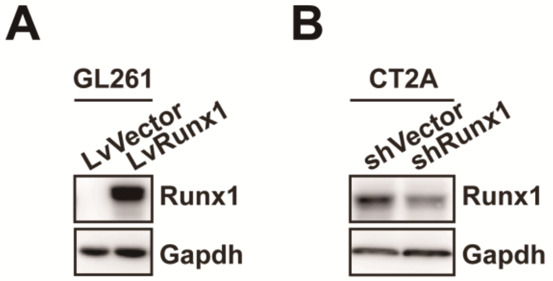

***Supplementary* Figure 6**

***Supplementary* Fig. S6, related to Fig. 6.** (**A-B**) WB analysis of Runx1 expression in Runx1 overexpressing GL261 and Runx1 downregulated CT2A cells. Gapdh was used as the loading control.

***Supplementary* Table S1. shRNA and siRNA sequences.**

Target	Sequence (5'-3')
shRUNX1-1	CCGGCCTCGAAGACATCGGCAGAAACTCGAGT TTCTGCCGATGTCTTCGAGGTTTTT
shRUNX1-3	CCGGTCGCCCTGTTTGGCATCTAATCTCGAGAT TAGATGCCAAACAGGGCGATTTTTG
shRunx1	CCGGCACCTACCATAGAGCCATCAACTCGAGTT GATGGCTCTATGGTAGGTGTTTTTG
siFOSL2-sense	GGCCCAGUGUGCAAGAUUAGC
siFOSL2-antisense	UAAUCUUGCACACUGGGCCGU
siNPM1-400-sense	GUGCAAAGGAUGAGUUGCATT
siNPM1-400-antisense	UGCAACUCAUCCUUUGCACTT
siNPM1-686-sense	GUAGCAAGGUUCCACAGAATT
siNPM1-686-antisense	UUCUGUGGAACCUUGCUACTT
siCBFβ-285-sense	GAAGCAAGUUCGAGAACGATT
siCBFβ-285-antisense	UCGUUCUCGAACUUGCUUCTT
siCBFβ-438-sense	CAGGAACCAAUCUGUCUCUTT
siCBFβ-438-antisense	AGAGACAGAUUGGUUCCUGTT

***Supplementary* Table S2. ChIP primer sequences.**

Gene	Sequence (5'-3')
ChIP-FN1-F	CAAGACAGTACATAGGGTGC
ChIP-FN1-R	GGAAAGTTGGACCAGCTGTG
ChIP-COL4A1-F	CATCAGGGCAGAACGATGTA
ChIP-COL4A1-R	CTGTCCCTCCTCTCCTTACT
ChIP-LUM-F	TACATTGTCTCAGCTCCATC
ChIP-LUM-R	CAGGAGTATAAATGGAGAGT

***Supplementary* Table S3. Descriptions of antibodies**

Antibody	Source	Identifier
H3K4me1	Cell Signaling Technology	5326S
H3K4me3	Cell Signaling Technology	9751S
H3K27ac	Cell Signaling Technology	8173S
H3K9me3	Cell Signaling Technology	13969S
FN1	Proteintech	66042-1-Ig
COL-IV	Proteintech	55131-1-AP
LUM	Abcam	ab168348
RUNX1	Abcam	ab23980
	Cell Signaling Technology	4336S
	Santa Cruz Biotechnology	sc365644
GAPDH	Proteintech	60004-1-Ig
β-Tubulin	Proteintech	66240-1-Ig
CBFβ	Abcam	ab125191
NPM1	Proteintech	10306-1-AP
FOSL2	Cell Signaling Technology	19967S
CD206	Cell Signaling Technology	24595S
MHC-II	Abcam	ab23990
CD16/32	BioLegend	101319
CD45.2	BioLegend	109806
CD3	BioLegend	100236
CD4	BioLegend	100408
CD8	BioLegend	100734

***Supplementary* Table S4. qRT-PCR primer sequences.**

Gene	Forward primer sequence (5'-3')	Reverse primer sequence (5'-3')
Human RUNX1	CCACCTACCACAGAGCCATCAA	TTCACTGAGCCGCTCGGAAAAG
Human FN1	ACAACACCGAGGTGACTGAGAC	GGACACAACGATGCTTCCTGAG
Human COL4A1	TGTTGACGGCTTACCTGGAGAC	GGTAGACCAACTCCAGGCTCTC
Human LUM	AACATACCAACTGTCAATGAAAACC	TGCCATCCAAACGCAAATGCTTG
Human GAPDH	GTCTCCTCTGACTTCAACAGCG	ACCACCCTGTTGCTGTAGCCAA
Human NPM1	GCCAGTGCATATTAGTGGACAGC	GGAACCTTGCTACCACCTCCAG
Human FOSL2	CAGAAATTCCGGGTAGATATGCC	GGTATGGGTTGGACATGGAGG
Mouse Runx1	CACCGTCTTTACAAATCCGCCAC	CGCTCGGAAAAGGACAAACTCC
Mouse Gapdh	CATCACTGCCACCCAGAAGACTG	ATGCCAGTGAGCTTCCCGTTCAG

Supplementary Table S5. WB original data.

Figure 2C

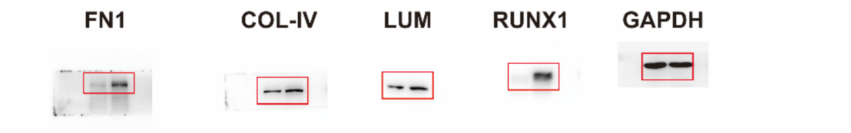

Figure 2D

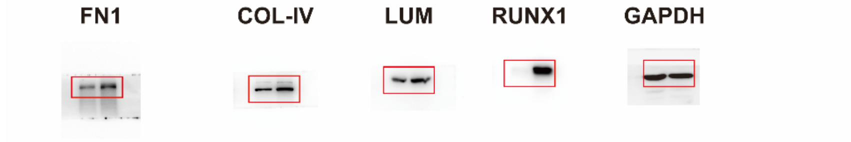

Figure 2I

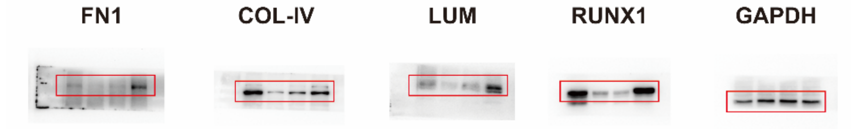

Figure 2J

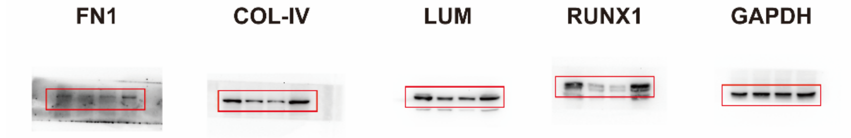

Supplementary Figure 2C

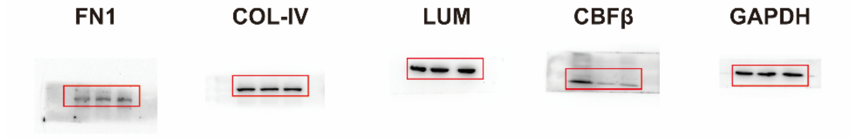

Supplementary Figure 2D

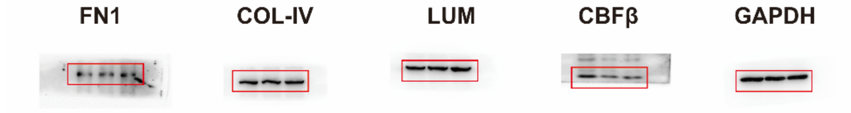

Supplementary Figure 2G

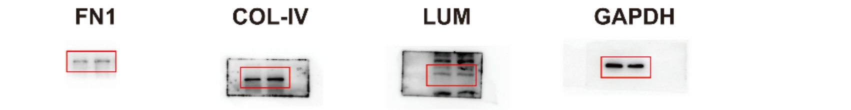

Supplementary Figure 2H

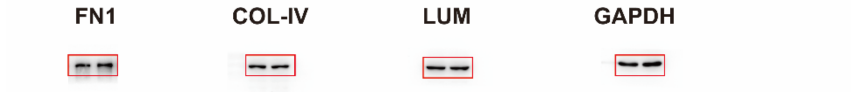

Figure 3B

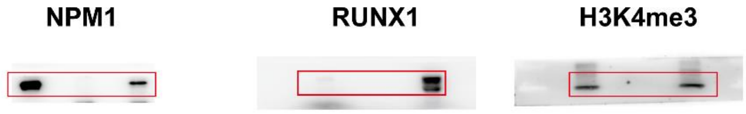

Figure 3C

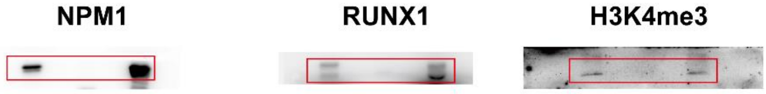

Figure 3F

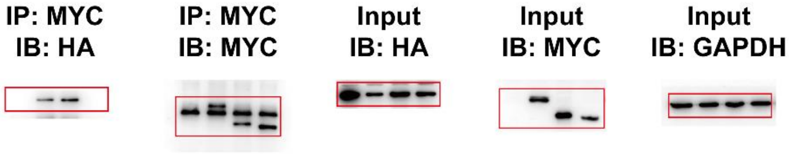

Figure 3G

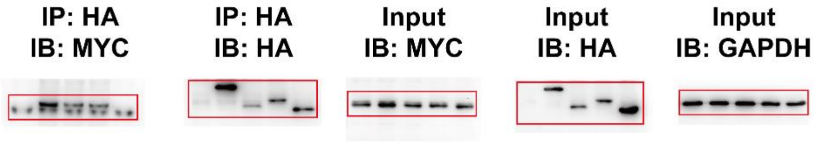

Figure 3J

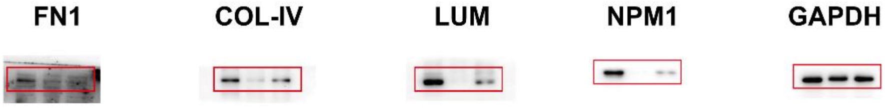

Figure 3K

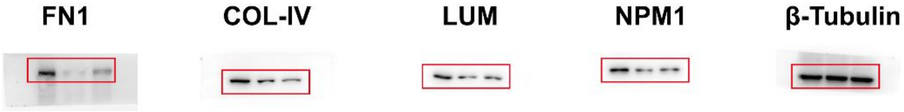

Figure 4H

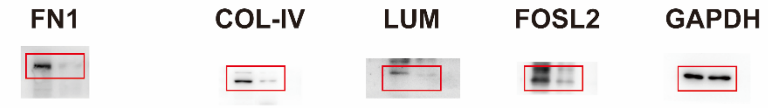

Figure 4K

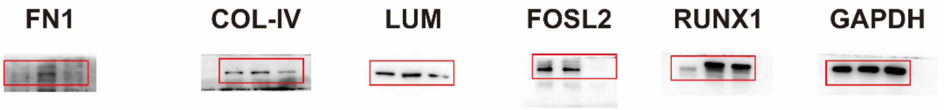

***Supplementary* Figure 4F**

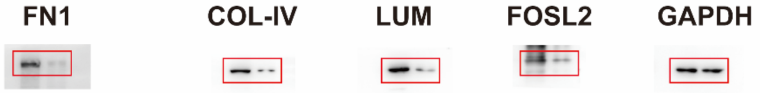

***Supplementary* Figure 4I**

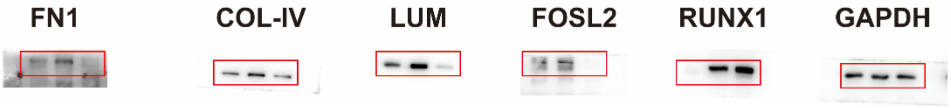

Supplement: Supplementary file 1 — Supplementary Materials [file 41419_2024_6481_MOESM1_ESM.pdf]
